# Improved Shear Strength Performance of Compacted Rubberized Clays Treated with Sodium Alginate Biopolymer

**DOI:** 10.3390/polym13050764

**Published:** 2021-02-28

**Authors:** Amin Soltani, Ramin Raeesi, Abbas Taheri, An Deng, Mehdi Mirzababaei

**Affiliations:** 1 School of Engineering, IT and Physical Sciences, Federation University, Churchill, VIC 3842, Australia; 2 Department of Infrastructure Engineering, The University of Melbourne, Parkville, VIC 3010, Australia; R.Raeesi@unimelb.edu.au; 3 School of Civil, Environmental and Mining Engineering, The University of Adelaide, Adelaide, SA 5005, Australia; Abbas.Taheri@adelaide.edu.au (A.T.); An.Deng@adelaide.edu.au (A.D.); 4 School of Engineering and Technology, Central Queensland University, Melbourne, VIC 3000, Australia; M.Mirzababaei@cqu.edu.au

**Keywords:** clay soil, ground rubber, sodium alginate, uniaxial compressive strength, scanning electron microscopy, curing duration, cationic bridging

## Abstract

This study examines the potential use of sodium alginate (SA) biopolymer as an environmentally sustainable agent for the stabilization of rubberized soil blends prepared using a high plasticity clay soil and tire-derived ground rubber (GR). The experimental program consisted of uniaxial compression and scanning electron microscopy (SEM) tests; the former was performed on three soil-GR blends (with GR-to-soil mass ratios of 0%, 5% and 10%) compacted (and cured for 1, 4, 7 and 14 d) employing distilled water and three SA solutions—prepared at SA-to-water (mass-to-volume) dosage ratios of 5, 10 and 15 g/L—as the compaction liquid. For any given GR content, the greater the SA dosage and/or the longer the curing duration, the higher the uniaxial compressive strength (UCS), with only minor added benefits beyond seven days of curing. This behavior was attributed to the formation and propagation of so-called “cationic bridges” (developed as a result of a “Ca^2+^/Mg^2+^ ⟷ Na^+^ cation exchange/substitution” process among the clay and SA components) between adjacent clay surfaces over time, inducing flocculation of the clay particles. This clay amending mechanism was further verified by means of representative SEM images. Finally, the addition of (and content increase in) GR—which translates to partially replacing the soil clay content with GR particles and hence reducing the number of available attraction sites for the SA molecules to form additional cationic bridges—was found to moderately offset the efficiency of SA treatment.

## 1. Introduction

The rapid economic development of modern societies has resulted in a dramatic rise in waste generation. Waste tires from the automotive industry, commonly referred to as end-of-life tires (or ELTs), are among the largest and most problematic of these waste streams and hence demand further attention. ELT stockpiles are often associated with harmful environmental impacts, particularly when subjected to improper waste management techniques—that is, occupancy of valuable landfill space (owing to the tires’ low mass-to-volume ratio), water stagnation and air pollution (on ignition) [1,2,3]. To alleviate these burdens on the environment, particularly the need for landfilling, local communities and governmental agencies have been increasingly persuaded to recycle and reuse ELTs, as a construction material, within their infrastructure systems (e.g., pavements, embankments, retaining walls and bridge abutments). Recycled ELT-based derivatives, with synthetic rubber being their main constituent, are resilient, light-weight and skin-resistive; these attributes make them one of the most suitable candidates for developing high-performance geomaterials for a variety of geotechnical engineering applications [4,5,6]. Recent research involving the addition of recycled ELT-based products—mainly in the form of granulates, herein referred to as ground rubber (GR)—to low-grade clay soils has shown that the compacted soil-GR blend demonstrates excellent properties in terms of diminishing the soil’s swell–shrink volume change capacity, as well as its desiccation-induced cracking potential [6,7,8,9,10,11,12,13,14,15]. Moreover, the application of GR-based additives has been reported to greatly enhance the soil’s damping, which adds greatly to its seismic resistance [16,17,18,19,20]. In terms of shear strength and bearing capacity, the addition of GR, at low GR-to-soil mass ratios (mainly less than 10%), has been found to produce relatively small improvements; however, soil-GR composites prepared with higher GR contents have been reported to create serious strength and stiffness concerns, and thus are generally not considered as high-performance geomaterials [6,9,11,13,21,22,23,24,25,26]. Accordingly, for those projects where the strength and stiffness of the composite geomaterial are primary concerns, the compacted soil-GR blend requires stabilization.

Much like natural soils, the stabilization of soil-GR blends can be accomplished using cementitious binders (e.g., Portland cement, hydrated lime, fly ash and silica fume). The introduction of these agents to the soil-GR–water complex results in the formation and propagation of a series of short- and long-term chemical reactions, which encourage flocculation of the soil and soil-GR constituents, thereby leading to major improvements in composite stiffness and shear strength [22,24,27,28,29]. It is well accepted that cementitious binders, even though effective in terms of stabilization, are generally not environmentally sustainable, since their application is often accompanied by significant energy and carbon emissions footprints [30,31,32]. This drawback alone highlights the urgency of minimizing the use of these binders in practice. A common solution in this context involves replacing these traditional binders, even though partially, with low-cost and more environmentally sustainable materials. Promising alternate materials capable of meeting both geotechnical performance and sustainability requirements, as reported in the research literature, include synthetic or natural polymers, resins and sulfonated oils [31,33,34,35,36,37,38,39,40,41].

Much like traditional cementitious binders, the introduction of polymers to the soil–water medium can encourage flocculation of the clay particles through various clay–polymer interaction mechanisms—that is, van der Waals or hydrogen bonding, charge neutralization (by way of electrostatic attraction) and cationic bridging for neutral, cationic and anionic polymers, respectively [42,43,44,45]. Among the multitude of commercially available polymer-based soil stabilizers, those derived from natural resources, commonly referred to as biopolymers, appear to possess a variety of promising soil-amendment features while outperforming synthetic variants in terms of sustainability, hence demanding further attention [32]. Sodium alginate (SA) is a water-soluble, linear polysaccharide derived from brown algae; it consists of two linked anionic monomers—that is, *β*-D-mannuronic acid (M) and *α*-L-guluronic acid (G) residues [46,47]. In terms of its polymeric structure, SA is composed of homo-polymeric regions of G-residues (G-blocks) and M-residues (M-blocks), interspersed with regions of mixed monomers or MG-blocks [48]. SA has been successfully employed within a variety of industries, including, most importantly, its widespread application as a thickener, a gelling agent and an emulsifier in the food industry [49,50,51]. In the geotechnical context, reported applications for SA have been limited to natural soils and include increasing soil compaction efficiency, enhancing soil shear strength/stiffness and soil seepage/erosion control [52,53,54,55,56,57,58,59]. Though promising, the results reported by these studies, particularly in the context of clay soil stabilization, are still limited (and somewhat inconsistent) to warrant SA as an *ad hoc* soil stabilization solution. With waste-based geomaterials, such as soil-GR blends, gaining ground as a viable construction material in practice, the need to re-evaluate the efficacy of traditional and emerging soil stabilization agents (including biopolymers) for these new geomaterials (particularly those containing non-soil components) arises as an inevitable necessity. To the authors’ knowledge, the outcomes of biopolymer treatment, including the use of SA as a compaction liquid, on the mechanical performance of soil-GR blends (as well as other waste-based geomaterials containing non-soil components) have not yet been investigated (nor understood), thus implying the need for further research to fully understand the true stabilization potentials and/or limitations of SA-based agents. In the authors’ view, the lack of such published data, among other factors, has hindered the acceptance of biopolymers, as additives, for widespread usage in ground improvement projects.

In view of the limited literature on employing SA in ground improvement practice, this experimental study investigates the application of an SA-based biopolymer as an environmentally sustainable agent for the stabilization of soil-GR blends (prepared using a high plasticity clay soil). The primary objectives were to examine the outcomes of SA treatment, considering the effects of both SA dosage and short-term curing duration, on the shear strength and microstructure properties of compacted soil-GR blends. The experimental program consisted of uniaxial compression and scanning electron microscopy (SEM) tests; the former was performed on three different soil-GR blends (with GR-to-soil mass ratios of 0%, 5% and 10%) compacted (and cured for 1, 4, 7 and 14 d) using distilled water and three different SA solutions—prepared at three different SA-to-water (mass-to-volume) dosage ratios of 5, 10 and 15 g/L—as the compaction liquid. Finally, the fundamental principles of soil chemistry, along with typical SEM images, were employed to identify and hence discuss the soil–SA and soil-GR–SA amending mechanisms.

## 2. Test Materials

### 2.1. Test Soil

The soil selected for this experimental investigation was a high-plasticity clay; its pertinent physical and mechanical properties were determined in accordance with relevant ASTM and Australian standards, and the results are summarized in Table 1.

**Figure 1:**
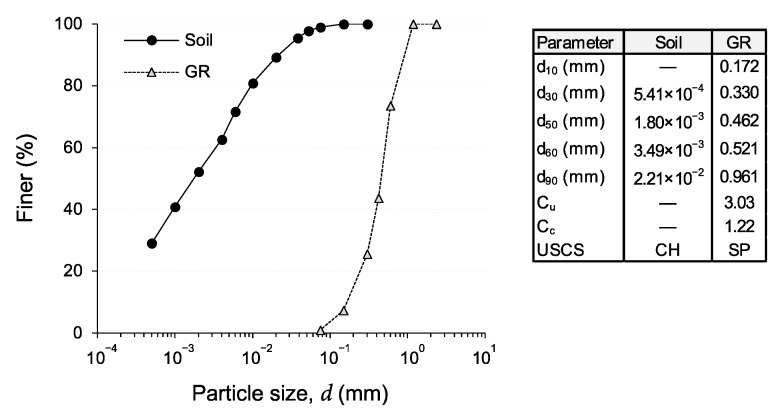
Particle-size distribution (PSD) curves for the test materials.

Referring to Figure 1, which illustrates the particle-size distribution (PSD) curves for the test materials; the test soil contained 1% fine sand (0.075–0.425 mm), 47% silt (2–75 μm) and 52% clay (<2 μm), dry mass basis as per ASTM D422 [60]. In terms of consistency, the fall-cone liquid limit (LL)—tested by means of a standard 80 g–30° fall-cone device in accordance with AS 1289.3.9.1 [61]—and the rolling-thread plastic limit (PL)—tested in accordance with AS 1289.3.2.1 [62]—were measured as LL = 84.3% and PL = 32.0% (mean value calculated based on three measurements, conducted by a single operator, with a standard deviation of 2.3%), thereby producing a plasticity index value of PI = LL − PL = 52.3%, as per AS 1289.3.3.1 [63]. Following the Unified Soil Classification System (USCS) outlined in ASTM D2487 [64], the test soil can be classified as clay with high plasticity (CH). Furthermore, the specific gravity of the soil solids was measured as $G_{s}^{S}$ = 2.73 (ASTM D854 [65]). In terms of compactability, the optimum moisture content (OMC) and maximum dry unit weight (MDUW)—tested under the standard Proctor energy (SPE) level (ASTM D698 [66])—were measured as *w*_opt_ = 28.0% and *γ*_dmax_ = 14.6 kN/m^3^, respectively; the latter is equivalent to a relatively high void ratio of *e* = 0.834.

### 2.2. Tire-Derived Ground Rubber

**Figure 2:**
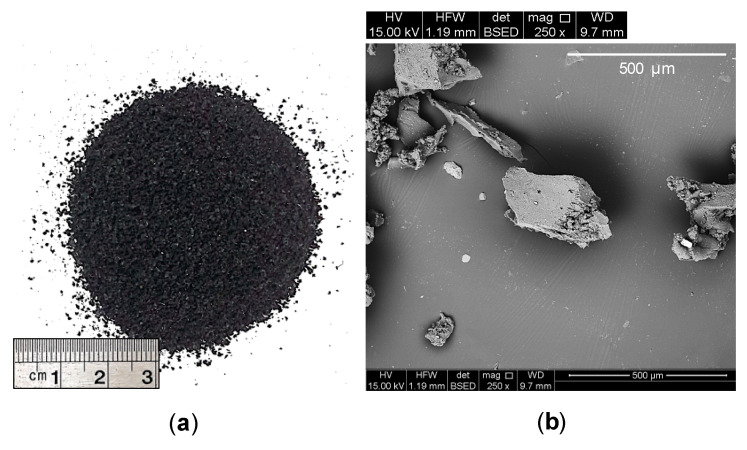
The GR material at different magnification ratios of (**a**) 1× and (**b**) 250×.

Commercially available tire-derived ground rubber (GR), sourced from a local distributor, was used to prepare rubberized clay blends (see Figure 2a). Referring to the PSD curves shown in Figure 1; the particles of the GR material were found to fall into the fine–medium sand (0.075–2 mm) gradational category, with its particle diameters corresponding to 10%, 30%, 50%, 60% and 90% finer being determined as *d*_10_ = 0.172 mm, *d*_30_ = 0.330 mm, *d*_50_ = 0.462 mm, *d*_60_ = 0.521 mm and *d*_90_ = 0.961 mm, respectively (ASTM D422 [60]). In view of these diameters, along with the calculated uniformity and curvature coefficients (i.e., *C*_u_ = *d*_60_/*d*_10_ = 3.03 and *C*_c_ = *d*_30_^2^/*d*_60_*d*_10_ = 1.22), the GR material can be classified as equivalent to poorly-graded sand (SP) in accordance with the USCS classification framework (ASTM D2487 [64]).

The scanning electron microscopy (SEM) technique was employed to observe the GR material’s morphological features (see Figure 2b). From visual inspection, the GR particles can be characterized as non-spherical and somewhat angular in shape, with occasional cavities and micro-cracks distributed along their surfaces, hence showcasing a rough surface texture [39,67]. Furthermore, as stated in the manufacturer’s literature, the GR material possessed a specific gravity of $G_{s}^{\mathrm{GR}}$ = 1.09 (i.e., 2.5-fold lower than that of the test soil with $G_{s}^{S}$ = 2.73), a specific surface area of 0.05 m^2^/g, and a softening point of 170 °C. In terms of average chemical composition, the GR material consisted of 55% styrene–butadiene copolymer, 25–35% carbon black, 5–20% acetone extract, 2–3% zinc oxide and 1–3% sulfur, mass basis.

### 2.3. Sodium Alginate Biopolymer

**Figure 3:**
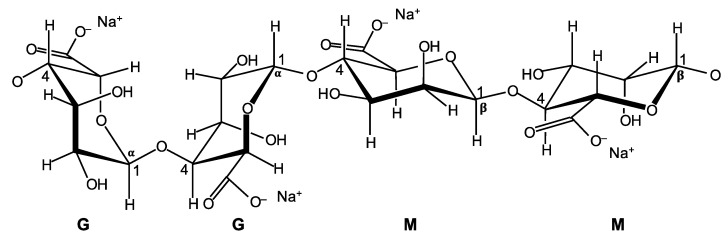
Chemical structure of SA (modified from [48]).

Commercially available SA, sourced from a local distributor, was used as the soil-GR-stabilizing agent. It was supplied in solid form (slightly yellow powder) and, as per the manufacturer’s instructions, is to be diluted with water for application. Referring to Figure 3; SAs consist of two linked anionic monomers, namely, *β*-D-mannuronic acid (M) and *α*-L-guluronic acid (G) residues, often structured as homo-polymeric regions of G-residues (G-blocks) and M-residues (M-blocks), interspersed with regions of mixed monomers or MG-blocks [48]. As reported in the manufacturer’s literature, the SA material used in this investigation possessed a molecular weight of 216 g/mol, a pH of 5.5–7.5 for a 1% aqueous solution (at 25 °C) and an average dynamic viscosity of 24.5 cP (1 cP = 10^−3^ Pa.s) for a 1% aqueous solution (at 25 °C).

## 3. Test Program

### 3.1. Mix Designs and Sample Preparations

The primary objectives of this experimental study were to investigate the outcomes of SA treatment, considering the effects of both SA dosage and short-term curing duration, on the shear strength and microstructure properties of soil-GR blends. Accordingly, a total of fourteen mix designs—including five untreated soil-GR blends and nine SA-treated soil-GR cases—were examined (see Table 2). For ease of presentation and analysis, herein, the following coding system employed to designate the investigated soil-GR–SA mix designs:

(1) $$R_{x}S_{y}T_{z}$$

where *R_x_* = *x*% GR (i.e., GR-to-soil mass ratio); *S_y_* = *y* g/L SA (i.e., SA-to-water mass-to-volume ratio); and *T_z_* = *z* days of curing.

It should be mentioned that, for those mix designs containing only GR, the GR material was incorporated into the test soil using four different GR contents of *f*_GR_ = 5%, 10%, 20% and 30%, with the GR content being defined by Equation (2). For SA-treated cases, the granular SA material was diluted with distilled water and applied as the mixing liquid employing three different SA dosages of *D*_SA_ = 5, 10 and 15 g/L, with the SA dosage being defined as per Equation (3). Moreover, the GR content for SA-treated cases was limited to *f*_GR_ ≤ 10%; as will be discussed in Section 4.1, GR contents greater than 10% can lead to serious shear strength concerns (under compression) and hence are unsuitable options for the development of high-performance rubberized soil blends.

(2) $$\left(\%\right)\;f_{\mathrm{GR}}=\frac{M_{\mathrm{GR}}}{M_{\mathrm{DS}}}\times 100$$

(3) $$\left(g/L\right)\;D_{\mathrm{SA}}=\frac{M_{\mathrm{SA}}}{V_{\mathrm{DW}}}$$

where *f*_GR_ = GR content (in %); *D*_SA_ = SA dosage (in g/L); *M*_DS_, *M*_GR_ and *M*_SA_ = mass of oven-dried soil, GR and granular SA, respectively (in g); and *V*_DW_ = volume of distilled water (in L).

Following the mix designs outlined in Table 2, the oven-dried test soil and GR material were first blended in dry form. On achieving visible homogeneity of the two ingredients, the required volume of liquid—either distilled water (for *R_x_S*_0_*T*_0_, where *x* = {0, 5, 10, 20, 30}) or SA solution (for *R_x_S_y_T_z_*, where *x* = {0, 5, 10}, *y* = {5, 10, 15} and *z* = {1, 4, 7, 14}) corresponding to the standard Proctor OMC value of various soil-GR blends (see *w*_o_ values in Table 2)—was added to each of the rubberized soil blends and thoroughly mixed by hand. Extensive care was taken (visually) to pulverize any clumped particles, particularly for higher SA dosages, thereby targeting homogeneity of the various test mixtures. As per common practice [6,13,39], samples for the uniaxial compression tests (see Section 3.2) were formed in a series of 50-mm diameter and 100-mm high stainless-steel molds in three equal-height layers by means of the static compaction technique, such that each layer was able to accomplish the standard Proctor MDUW value of the respective soil-GR blend (see *γ*_do_ values in Table 2). To assure adequate bonding between adjacent layers of the compacted samples, the surface of the first and second compacted layers was scarified. Furthermore, for those mix designs containing SA, four short-term curing durations of *T*_c_ = 1, 4, 7 and 14 d were considered, whereby the compacted samples were sealed (using multiple layers of cling wrap) and maintained at ambient laboratory conditions prior to uniaxial compression testing. Another point worth noting is that the specific gravity for the various soil-GR blends was estimated by the following theoretical relationship [8,68]:

(4) $$G_{s}^{\mathrm{mix}}=\frac{G_{s}^{S}G_{s}^{\mathrm{GR}}\left(M_{\mathrm{DS}}+M_{\mathrm{GR}}\right)}{G_{s}^{S}M_{\mathrm{GR}}+G_{s}^{\mathrm{GR}}M_{\mathrm{DS}}}$$

where $G_{s}^{\mathrm{mix}}$ = soil-GR mixture specific gravity (values presented in Table 2); *M*_DS_ and *M*_GR_ = mass of oven-dried soil and GR, respectively (in g); and $G_{s}^{S}$ and $G_{s}^{\mathrm{GR}}$ = specific gravity of soil solids and GR particles (= 2.73 and 1.09), respectively.

**Figure 4:**
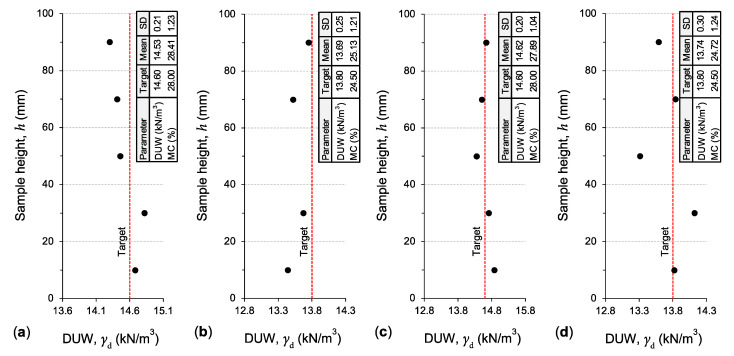
Variations of the DUW parameter along the height of representative SPE-compacted samples: (**a**) *R*_0_*S*_0_*T*_0_; (**b**) *R*_10_*S*_0_*T*_0_; (**c**) *R*_0_*S*_15_*T*_0_; and (**d**) *R*_10_*S*_15_*T*_0_.

To ensure that the compacted samples were uniform in fabric (i.e., blending homogeneity) and hence repeatable in behavior, on completion of static compaction, the variations of both the DUW and MC parameters were measured along the height of representative samples, namely, *R*_0_*S*_0_*T*_0_, *R*_10_*S*_0_*T*_0_, *R*_0_*S*_15_*T*_0_ and *R*_10_*S*_15_*T*_0_. As per common practice [69,70,71], this was achieved by slicing each of the aforementioned compacted samples into five equal-height segments and testing each segment for DUW and MC, as illustrated in Figure 4. The variations of both the DUW and MC parameters were found to be marginal, as supported by the low standard deviation (SD) values (e.g., 0.2 kN/m^3^ ≤ SD ≤ 0.3 kN/m^3^ for DUW), thus confirming the suitability and repeatability of the employed sample preparation technique (in terms of mixing and compaction).

### 3.2. Uniaxial Compression Test

Uniaxial compression tests were performed following ASTM D2166 [72]. The prepared SPE-compacted samples (prepared as per Section 3.1) were axially compressed using a constant displacement rate of 1 mm/min (equivalent to an axial strain rate of 1%/min), as commonly employed for testing GR mixed fine-grained soils [6,10,67]. For each sample, the axial strain and corresponding mobilized axial stress were measured (using a closed-loop servo-controlled hydraulic compressive machine with a maximum load capacity of 50 kN) at predefined time intervals until such time that the ultimate/peak axial stress (required for sample failure)—defined as the uniaxial compressive strength (UCS)—was fully mobilized.

On account of the four curing durations (i.e., *T*_c_ = 1, 4, 7 and 14 d) adopted for SA-treated samples, a total of 41 uniaxial compression tests—that is, 5 for the untreated soil-GR blends and 36 for the SA-treated soil-GR mixtures—were conducted to address the fourteen mix designs outlined in Table 2. To assure sufficient accuracy, triplicate samples were tested for representative mix designs, namely, *R*_0_*S*_0_*T*_0_, *R*_10_*S*_0_*T*_0_, *R*_0_*S*_15_*T*_7_ and *R*_10_*S*_15_*T*_7_, with the coefficient of variation (CV) for the UCS parameter being calculated as CV = 3.4%, 5.7%, 3.8% and 4.9%, respectively. These low CV values corroborate the repeatability of the adopted sample preparation technique, as well as the implemented uniaxial compression testing procedure.

### 3.3. Scanning Electron Microscopy Test

Representative samples, including *R*_0_*S*_0_*T*_0_, *R*_10_*S*_0_*T*_0_, *R*_0_*S*_10_*T*_7_ and *R*_10_*S*_10_*T*_7_, were investigated using the SEM technique to observe the evolution of soil fabric due to the addition of GR and/or SA. On completion of curing (if applicable), the aforementioned SPE-compacted samples (prepared as per Section 3.1) were first carefully fractured into small cubic-shaped specimens (measuring approximately 1 cm^3^ in volume). These specimens were allowed to air-dry for approximately fourteen days; the air-dried specimens were then scanned at various magnification ratios ranging between 150× and 20,000×. It should be mentioned that the Philips XL20 (Koninklijke Philips N.V., Amsterdam, The Netherlands) scanning electron microscope was employed for SEM imaging. Its main specifications, as reported in the manufacturer’s literature, included a resolution of 4 μm and a maximum magnification ratio of 50,000×.

## 4. Results and Discussion

### 4.1. Effects of Ground Rubber on Soil Compactability and Compressive Strength

The complete results of the standard Proctor compaction and uniaxial compression tests for the untreated soil-GR blends are summarized in Table 3. The untreated soil-GR mixtures—that is, *R*_5_*S*_0_*T*_0_, *R*_10_*S*_0_*T*_0_, *R*_20_*S*_0_*T*_0_ and *R*_30_*S*_0_*T*_0_—were all found to produce lower OMC and MDUW values compared with those obtained for the test soil (*R*_0_*S*_0_*T*_0_). In this regard, the greater the GR content, the lower the OMC and MDUW parameters, both following monotonically decreasing trends. The OMC and MDUW parameters for the test soil (*R*_0_*S*_0_*T*_0_) were measured as *w*_opt_ = 28.0% and *γ*_dmax_ = 14.6 kN/m^3^, respectively. With the addition of 5%, 10%, 20% and 30% GR (*R*_5_*S*_0_*T*_0_, *R*_10_*S*_0_*T*_0_, *R*_20_*S*_0_*T*_0_ and *R*_30_*S*_0_*T*_0_), the aforementioned values decreased to *w*_opt_ = 26.2%, 24.5%, 22.2% and 20.6%, and *γ*_dmax_ = 14.3, 13.8, 13.4 and 12.9 kN/m^3^, respectively.

The observed reductions in the OMC parameter can be attributed to the GR material’s hydrophobic character and hence lower water adsorption–retention capacity compared with that of the clay soil particles [10,73]. As for the MDUW parameter, the GR material’s lower specific gravity (of $G_{s}^{\mathrm{GR}}$ = 1.09) compared to that of the soil solids (with $G_{s}^{S}$ = 2.73) elucidates the lower MDUW values observed for the various soil-GR mixtures [22]. Moreover, because of their high energy absorption capacity, the compacted GR particles may progressively recover their initial (or uncompacted) shapes by way of a so-called “elastic rebound” mechanism, thereby reducing the efficiency of the imposed compaction energy and hence yielding lower MDUW values [67,68]. With this in mind, one can postulate that, the greater the GR content, the higher the energy absorption capacity of the soil-GR mixture and hence the more pronounced the mixture’s overall elastic-rebound recovery.

Referring to Table 3; in terms of strength development, the variations of the UCS parameter with respect to GR content (i.e., the *q*_u_–*f*_GR_ relationship, where 0% ≤ *f*_GR_ ≤ 30%) demonstrated a rise–fall trend, peaking at *f*_GR_ = 5% and then decreasing for higher GR contents, such that the samples containing 20% and 30% GR exhibited noticeably lower UCS values compared with that obtained for the test soil. The test soil (*R*_0_*S*_0_*T*_0_) and its various GR-blended samples containing 5%, 10%, 20% and 30% GR (*R*_5_*S*_0_*T*_0_, *R*_10_*S*_0_*T*_0_, *R*_20_*S*_0_*T*_0_ and *R*_30_*S*_0_*T*_0_) produced UCS values of *q*_u_ = 127.6, 146.4, 120.5, 115.4 and 81.3 kPa, respectively. These results can be discussed at the soil-GR agglomeration-scale level as follows.

At the soil-GR agglomeration-scale level, where the GR particles can be portrayed as embedded coarse-grained isolated solid inclusions, the UCS capacity of compacted soil-GR blends is provided by the combination of two resistive forces—namely, the cohesion resistance of its soil solids matrix (soil–soil interface), and an adhesion resistance generated at the surfaces of the isolated embedded GR particles (soil-GR interface) [6]. On account of the adhesion resistance being noticeably lower than the cohesion resistance, compared to the overall cohesion resistance of an individual soil agglomeration containing no GR, the combined cohesion–adhesion resistances for the soil-GR agglomeration would be lower overall and are expected to progressively decrease with increasing the GR content—in other words, the UCS should normally decrease with the addition of (and content increase in) GR, hence explaining the lower UCS values of the samples containing *f*_GR_ = 10%, 20% and 30% (*R*_10_*S*_0_*T*_0_, *R*_20_*S*_0_*T*_0_ and *R*_30_*S*_0_*T*_0_) compared with that mobilized for the test soil (*R*_0_*S*_0_*T*_0_). For the sample containing 5% GR (*R*_5_*S*_0_*T*_0_), where the mobilized UCS was found to be greater than that of the test soil, arching between the GR inclusions within the soil-GR agglomerations can be considered as the governing mechanism [6]. In general, the greater the GR content, the more pronounced the positive effects of arching and hence the higher the developed UCS. With this in mind, one can postulate that, for the compacted sample containing *f*_GR_ = 5%, the positive effects of arching were still dominant compared to the negative effects of the cohesion–adhesion mechanism described above and, as such, the UCS was able to increase beyond the test soil. Accordingly, for GR contents greater than 5%, where the mobilized UCS dropped below that of the test soil, the negative effects of the cohesion–adhesion mechanism, particularly for *f*_GR_ = 20% and 30%, were able to prevail against arching, even though the positive effects of arching were greater compared with 5% GR. It is also worth mentioning that the GR particles themselves possess greater deformability (or lower rigidity) compared with that of the soil solids—that is, the soil-GR agglomerations possess substantially lower stiffness compared with that of the individual soil agglomerations containing no GR [5,67,74]. This notable mismatch in relative stiffness, particularly for GR contents greater than 10%, gives the soil-GR agglomerations a so-called “friable” nature, such that in unconfined compression, the GR-blended samples undergo greater radial expansion and hence mobilize lower UCS values [6].

### 4.2. Combined Effects of Ground Rubber and Sodium Alginate on Soil Compressive Strength

**Figure 5:**
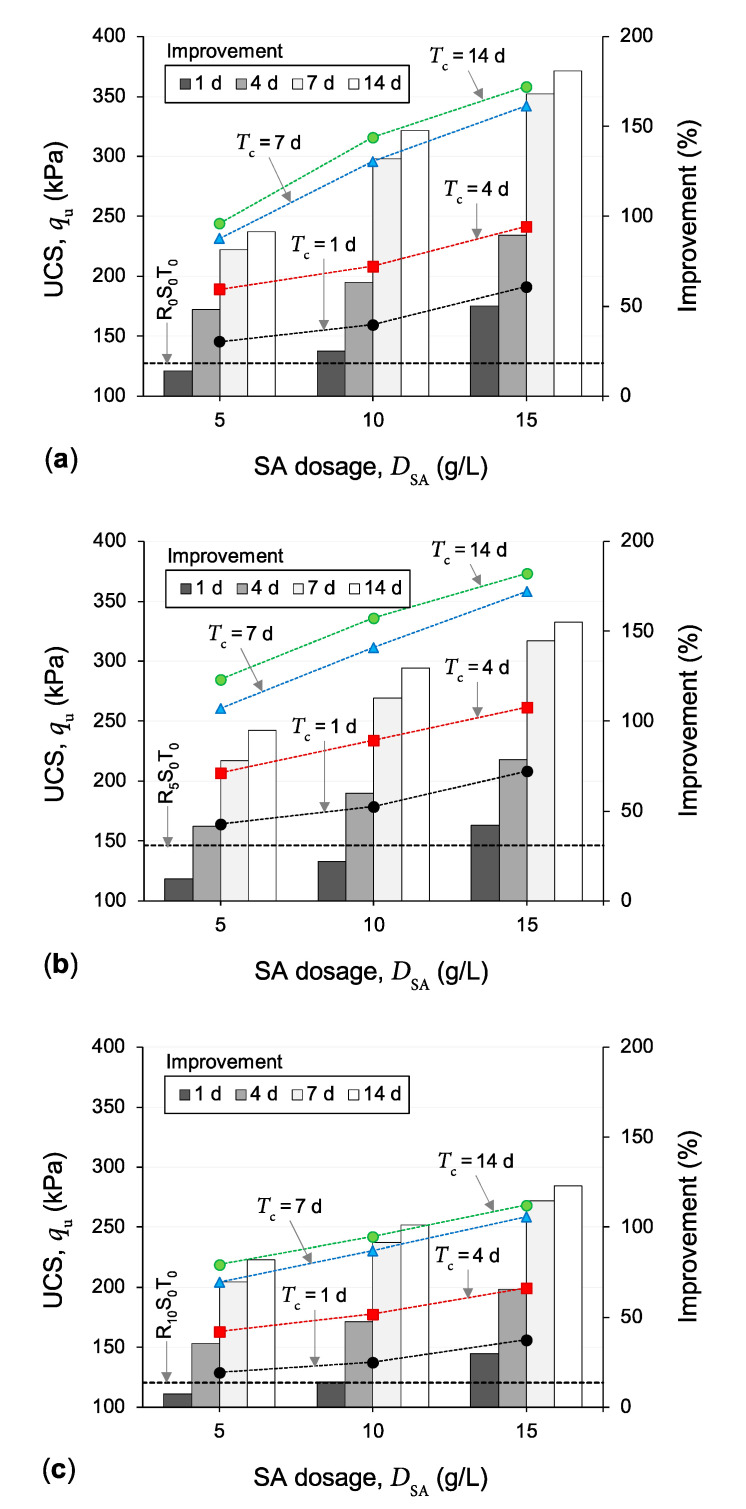
Variations of the UCS parameter against SA dosage for the samples (**a**) *R*_0_*S_y_T_z_*, (**b**) *R*_5_*S_y_T_z_* and (**c**) *R*_10_*S_y_T_z_*, where *y* = {5, 10, 15} and *z* = {1, 4, 7, 14}.

Figure 5a–c illustrate the variations of the UCS parameter against SA dosage for the samples *R*_0_*S_y_T_z_*, *R*_5_*S_y_T_z_* and *R*_10_*S_y_T_z_*, where *y* = {5, 10, 15} and *z* = {1, 4, 7, 14}, respectively. For any given GR content and curing duration, the greater the employed SA dosage, the higher the mobilized UCS, following a monotonically increasing trend. For instance, the untreated sample containing 5% GR (*R*_5_*S*_0_*T*_0_) produced a UCS value of 146.4 kPa; meanwhile, the same sample treated with *D*_SA_ = 5, 10 and 15 g/L and cured for *T*_c_ = 7 d (*R*_5_*S*_5_*T*_7_, *R*_5_*S*_10_*T*_7_ and *R*_5_*S*_15_*T*_7_) resulted in higher UCS values of *q*_u_ = 260.7, 311.5 and 358.3 kPa (i.e., equivalent to improvements of 78%, 113% and 145% in relation to *R*_5_*S*_0_*T*_0_), respectively. Moreover, for any given GR content and SA dosage, an increase in curing duration led to a notable increase in the UCS parameter up to *T*_c_ = 7 d, albeit often with a noticeably higher incremental rate compared with that achieved by increasing the SA dosage (for the investigated ranges of 5 g/L ≤ *D*_SA_ ≤ 15 g/L and 1 d ≤ *T*_c_ ≤ 14 d). Beyond seven days of curing, the positive effects of curing became less pronounced and, in most cases, rather marginal. As typical cases highlighting the effects of curing duration (for a constant GR content and SA dosage), the samples *R*_5_*S*_10_*T*_1_, *R*_5_*S*_10_*T*_4_, *R*_5_*S*_10_*T*_7_ and *R*_5_*S*_10_*T*_14_ resulted in *q*_u_ = 178.8, 234.1, 311.5 and 336.2 kPa (i.e., equivalent to improvements of 22%, 60%, 113% and 130% in relation to *R*_5_*S*_0_*T*_0_), respectively.

It is also worth mentioning that, for any given SA dosage and curing duration, the UCS–GR content (or *q*_u_–*f*_GR_) relationship (where 0% ≤ *f*_GR_ ≤ 10%) exhibited a rise–fall trend (similar to that discussed in Section 4.1 for the untreated soil-GR samples), peaking at *f*_GR_ = 5% and then falling below its 0%-GR counterpart for *f*_GR_ = 10%. For instance, the samples treated with 10 g/L SA and cured for seven days produced UCS values of 296.1, 311.5 and 230.6 kPa for *f*_GR_ = 0%, 5% and 10%, respectively.

**Figure 6:**
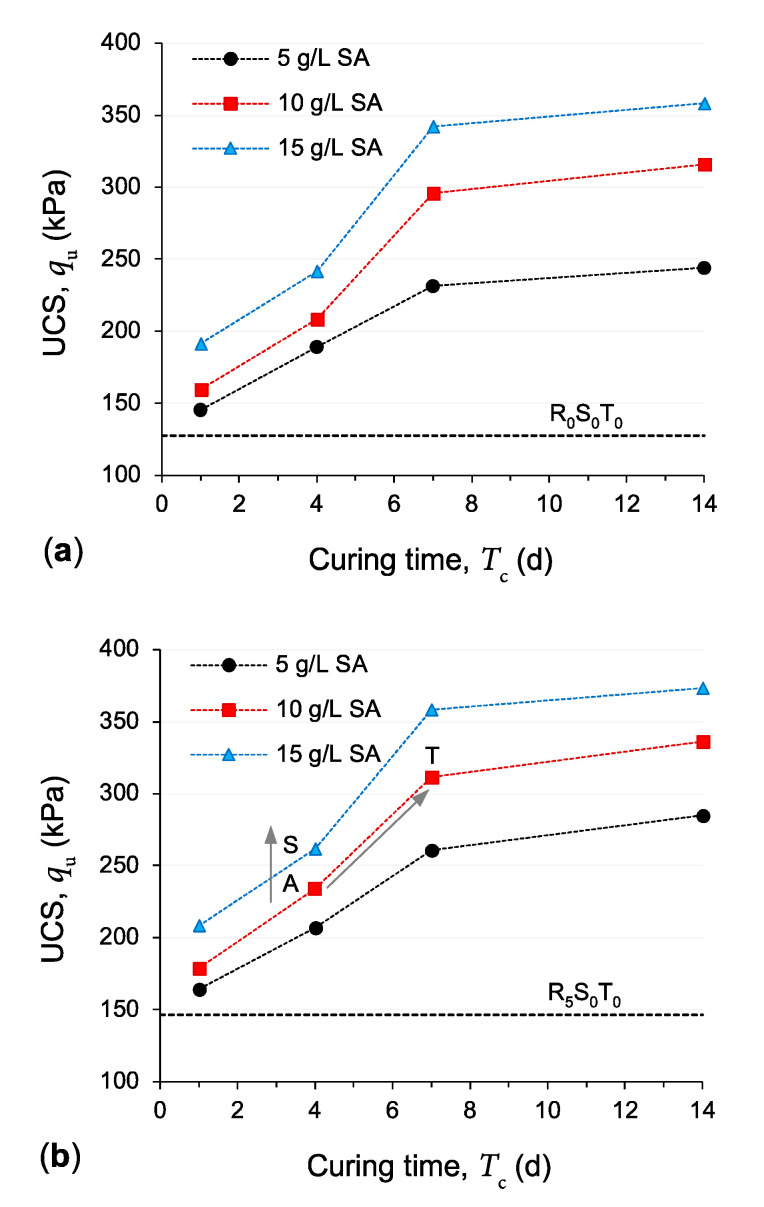

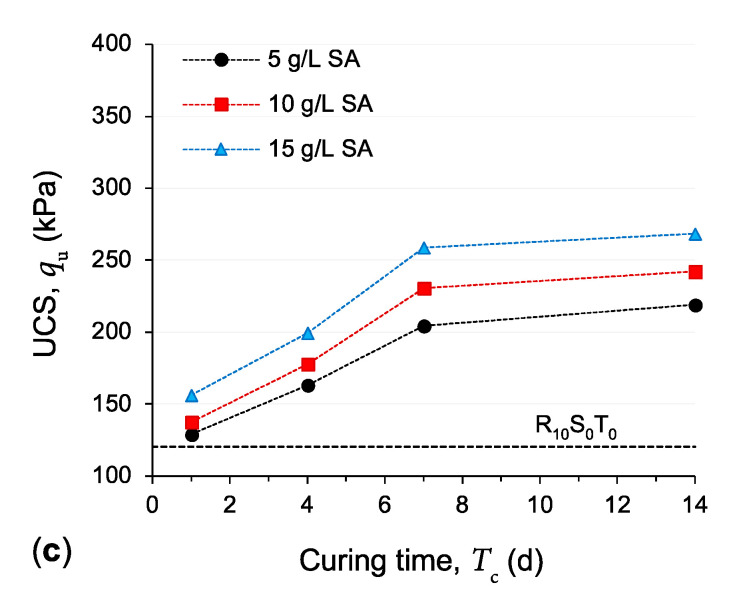
Variations of the UCS parameter against curing duration for the samples (**a**) *R*_0_*S_y_T_z_*, (**b**) *R*_5_*S_y_T_z_* and (**c**) *R*_10_*S_y_T_z_*, where *y* = {5, 10, 15} and *z* = {1, 4, 7, 14}.

Further highlighting the effects of curing duration; Figure 6a–c illustrate the variations of the UCS parameter against curing duration for the samples containing *f*_GR_ = 0%, 5% and 10%, respectively. For any given GR content and SA dosage, the mobilized UCS was found to follow an exponentially increasing trend with increasing the curing duration; only minor added improvements were noted beyond seven days of curing. Interestingly, the overall positive contribution to the UCS parameter provided by increasing the SA dosage (for the investigated range of 5 g/L ≤ *D*_SA_ ≤ 15 g/L) was found to be somewhat less prominent compared with that offered by extending the curing duration (particularly for *T*_c_ < 7 d)—that is, for a given increase in the duration of curing, the magnitude of improvement in the mobilized UCS was often greater than that achieved for a given increase in the SA dosage; as such, any reduction in the UCS imposed by a decrease in the SA dosage may be effectively moderated by increasing the curing duration. For instance, for the sample *R*_5_*S*_10_*T*_4_ (see point “A” in Figure 6b), a 5 g/L increase in SA dosage (i.e., *R*_5_*S***_10_***T*_4_ ⟶ *R*_5_*S***_15_***T*_4_) produced a 12% improvement in the mobilized UCS (follow the path “A ⟶ S” in Figure 6b); meanwhile, a three-day extension in curing for the same sample (i.e., *R*_5_*S*_10_*T***_4_** ⟶ *R*_5_*S*_10_*T***_7_**) resulted in a noticeably higher improvement of 33% (follow the path “A ⟶ T” in Figure 6b).

To investigate the effects of GR content on the efficiency of SA treatment, a new parameter, referred to as the strength-gain-factor (SGF), was defined as follows:

(5) $$\mathrm{SGF}=\frac{q_{u}^{T}}{q_{u}^{U}}$$

where SGF = strength-gain-factor; $q_{u}^{U}$ = UCS of untreated soil-GR samples—that is, *R*_0_*S*_0_*T*_0_, *R*_5_*S*_0_*T*_0_ or *R*_10_*S*_0_*T*_0_; and $q_{u}^{T}$ = UCS of SA-treated soil-GR samples—that is, *R*_0_*S_y_T_z_*, *R*_5_*S_y_T_z_* or *R*_10_*S_y_T_z_*, where *y* = {5, 10, 15} and *z* = {1, 4, 7, 14}.

**Figure 7:**
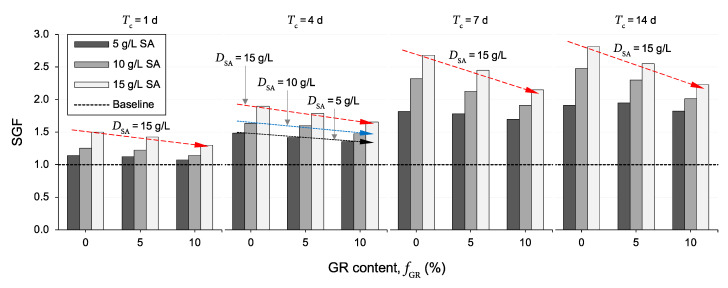
Variations of the SGF parameter—defined as the UCS ratio of an SA-treated sample to its non-treated counterpart—against GR content for the tested samples.

Figure 7 illustrates the variations of the SGF parameter—that is, the UCS ratio of an SA-treated sample to its non-treated counterpart, as defined in Equation (5)—against GR content for the tested samples. As is evident from this figure, for any given SA dosage and curing duration, an increase in GR content was accompanied by a somewhat notable reduction in the SGF parameter, particularly for *f*_GR_ = 10%—for further reference, follow the three trendlines labeled as “*D*_SA_ = 5 g/L, *D*_SA_ = 10 g/L and *D*_SA_ = 15 g/L” for *T*_c_ = 4 d. For instance, the samples *R*_0_*S*_10_*T*_4_, *R*_5_*S*_10_*T*_4_ and *R*_10_*S*_10_*T*_4_ produced SGF values of 1.63, 1.60 and 1.47, respectively. Moreover, the rate of reduction in the SGF parameter (with respect to GR content) was found to be more pronounced for higher SA dosages and/or longer curing durations—for instance, compare the three trendlines labeled as “*D*_SA_ = 15 g/L” for *T*_c_ = 1, 4, 7 and 14 d; these trendlines produced reduction rates (i.e., trendline slopes) of ΔSGF/Δ*f*_GR_ = −0.10, −0.12, −0.27 and −0.29, respectively.

The observed improvements in the UCS parameter, as achieved by SA treatment for the investigated compacted samples, can be discussed in the context of clay–SA interactions. In the presence of water, the divalent cations present in the vicinity of the negatively charged clay surfaces, including calcium (Ca^2+^) and magnesium (Mg^2+^), tend to substitute the lower-valance sodium cations (Na^+^) of the SA molecules, with Ca^2+^ initiating the substitution process and first replacing Na^+^, owing to its larger ionic radius compared to that of Mg^2+^ [54,57,58,75]. Since these divalent cations would still remain electrostatically attracted to the negatively charged clay surfaces, the so-called “cation exchange/substitution” process described above allows the SA molecules to be attracted (and hence adsorbed) to the negatively charged clay surfaces—in other words, the exchanged divalent cations function as so-called “attraction bridges” between the clay particles and SA molecules [42,44,54]. The formation and propagation (over time) of these strong cationic bridges between adjacent clay surfaces—which bring and hold the clay particles together—induce flocculation of the clay particles, thus increasing the soil’s overall shear resistance (and hence its UCS capacity). Provided that the number of attraction sites—that is, the number of clay particles (or the soil clay content) and/or the amount of exchangeable divalent cations (i.e., Ca^2+^ and Mg^2+^)—available for the SA molecules is not exhausted, one can postulate that, the greater the SA dosage, the higher the propensity for clay particle flocculation and hence the higher the mobilized UCS [39,41,76]; this mechanism explains the monotonically increasing trend observed for the UCS with increasing the SA dosage (see Figure 5). With this in mind, for any given SA dosage and curing duration, the addition of (and content increase in) GR—which translates to partially replacing the soil clay content with GR particles and hence reducing the number of available attraction sites for the SA molecules—is expected to decrease the magnitude of improvement in the UCS compared with that achieved for the test soil containing no GR (see Figure 7). An important by-product of the cation substitution process described above is the formation of hydrogel materials—that is, a network of cross-linked polymer chains which are hydrophilic in nature [57,58,77,78]. The formation, diffusion and hardening (due to hydration) of these hydrogel materials over time can be visualized as a three-dimensional reinforcement network acting in favor of weaving/interlocking the soil–soil and soil-GR agglomerations into a coherent matrix of improved shear strength performance, thus explaining the positive effects of curing on increasing the UCS (see Figure 6). It is worth mentioning that the possible exchange of hydrated inorganic cations in the interlayer for some types of organic cations is also important; such modified materials, however, possess a hydrophobic character and are suitable as components in clay–polymer nanocomposites [79].

### 4.3. Microstructure Analysis

**Figure 8:**
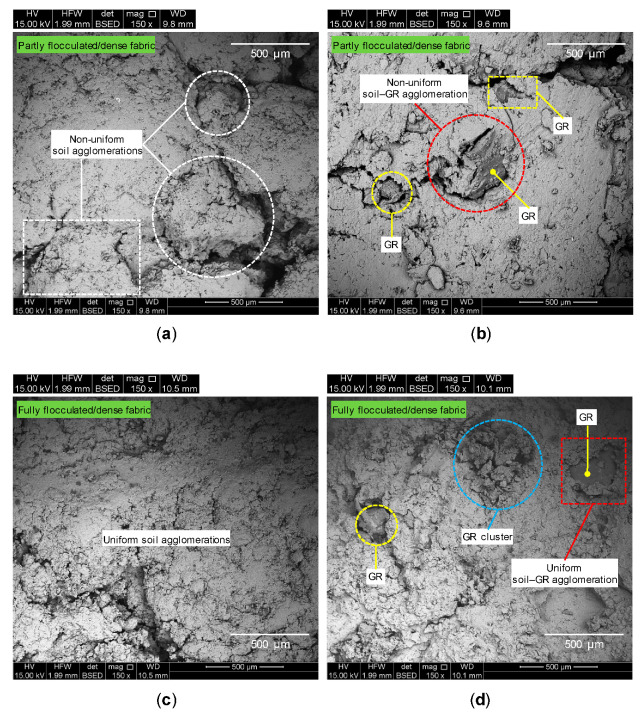
SEM micrographs for the tested samples: (**a**) *R*_0_*S*_0_*T*_0_; (**b**) *R*_10_*S*_0_*T*_0_; (**c**) *R*_0_*S*_10_*T*_7_; and (**d**) *R*_10_*S*_10_*T*_7_.

The microstructure analysis was performed employing an SEM characterization scheme developed by Soltani et al. [80]. Figure 8a–d illustrate SEM micrographs for the samples *R*_0_*S*_0_*T*_0_, *R*_10_*S*_0_*T*_0_, *R*_0_*S*_10_*T*_7_ and *R*_10_*S*_10_*T*_7_, respectively. Referring to Figure 8a; the microstructure of the compacted test soil containing no additives (*R*_0_*S*_0_*T*_0_) exhibited a highly porous structure, accompanied by a notable number of large inter- and intra-assemblage pore-spaces, respectively, formed between and within the soil agglomerations (or clay flocs). Such morphological features confirm the existence of a partly flocculated/dense fabric. The microstructure of the sample containing only 10% GR (*R*_10_*S*_0_*T*_0_) inherited the same highly porous and irregular structure of the test soil (*R*_0_*S*_0_*T*_0_), with the randomly distributed GR particles—visualized as embedded coarse-grained “isolated solid inclusions”—further contributing towards fabric deflocculation (see Figure 8b).

As a result of SA treatment (see the sample *R*_0_*S*_10_*T*_7_ in Figure 8c), the inter- and intra-assemblage pore-spaces exhibited a notable reduction in both number and size, allowing the development of relatively larger and more uniform soil agglomerations compared with those of the test soil (*R*_0_*S*_0_*T*_0_). Such attributes predicate the existence of a fully flocculated/dense fabric, thereby confirming the postulated “flocculation–reinforcement” clay–SA stabilization mechanisms discussed in Section 4.2. Similarly, the concurrent use of GR and SA (see the sample *R*_10_*S*_10_*T*_7_ in Figure 8d) produced a more flocculated fabric compared with the same GR inclusion containing no SA treatment (*R*_10_*S*_0_*T*_0_). The soil-GR connection/bonding interfaces also appear to be improved, potentially enhancing the stability of the soil-GR agglomerations. Another interesting observation with regard to Figure 8d is the appearance of a relatively large GR cluster in the fabric; compared with the soil-GR agglomerations, the GR clusters are more friable in nature. With this in mind, one can postulate that, with the addition of (and, more importantly, content increase in) GR, the UCS response at some points within the GR-blended sample (either with or without SA) may be governed by a dominant, undesirable GR-to-GR particles’ interaction mechanism [4,22,67]. This additional factor may also explain the observed reductions in the samples’ UCS and SGF parameters with increasing the GR content (as discussed in Table 3 and Figure 7, respectively).

It should be mentioned that the original (or as-compacted) shape and extent of the inter- and intra-assemblage pore-spaces may have changed during the SEM specimen fabrication process (see Section 3.3), owing to sample fracturing and, more importantly, the development of tensile stresses (and hence micro-cracks) within the soil fabric during the fourteen-day desiccation process [80]. However, given that the SEM specimen fabrication process for all of the investigated mix designs was identical in terms of drying conditions (e.g., temperature, humidity and duration), the SEM micrographs presented in Figure 8, which are essentially relative in nature, still provide a rather reliable basis for the comparison of the four different mix designs. Additional tests employing the environmental SEM technique, which enables wet specimens to be observed through the use of partial water vapor pressure in the microscope specimen chamber, should be performed to further improve upon the authors’ postulated microstructure analysis.

## 5. Conclusions

This study examined the potential use of an SA-based biopolymer as an environmentally sustainable agent for the stabilization of soil-GR blends (prepared using a high plasticity clay soil). The experimental program consisted of uniaxial compression and SEM tests; the former was performed on three different soil-GR blends (with GR-to-soil mass ratios of *f*_GR_ = 0%, 5% and 10%) compacted (and cured for *T*_c_ = 1, 4, 7 and 14 d) using distilled water and three different SA solutions—prepared at three different SA-to-water (mass-to-volume) dosage ratios of *D*_SA_ = 5, 10 and 15 g/L—as the compaction liquid. In view of the experimental results and their interpretation, the following conclusions can be drawn from this study:

- For any given GR content and curing duration, the greater the employed SA dosage, the higher the mobilized UCS, following a monotonically increasing trend. This behavior was attributed to the formation and propagation of so-called strong “cationic bridges” (developed as a result of a “Ca^2+^/Mg^2+^ ⟷ Na^+^ cation exchange/substitution” process among the clay and SA components) between adjacent clay surfaces over time, which induce flocculation of the clay particles and hence increase the sample’s overall shear resistance (i.e., UCS capacity). This flocculation mechanism, and hence the cationic bridging assertion, was further discussed and validated by means of typical SEM images.
- Further, for any given GR content and SA dosage, the mobilized UCS was found to follow an exponentially increasing trend with an increasing curing duration; beyond seven days of curing, however, the positive effects of curing became less pronounced and, in most cases, rather marginal. Interestingly, the overall positive contribution to the UCS parameter provided by increasing the SA dosage (for the investigated SA dosage range) was found to be somewhat less prominent compared with that offered by extending the curing duration (particularly for *T*_c_ < 7 d).
- Finally, for any given SA dosage and curing duration, the variations of the UCS parameter with respect to GR content demonstrated a rise–fall trend, peaking at *f*_GR_ = 5% and then falling below its 0%-GR counterpart for 10% GR. An increase in GR content for any given SA dosage and curing duration was accompanied by a somewhat notable reduction in the samples’ SGF—that is, the UCS ratio of an SA-treated sample to its non-treated counterpart. This behavior was attributed to the partial replacement of the soil clay content with GR particles, reducing the number of available attraction sites (or clay surfaces) for the SA molecules to form additional cationic bridges.

## 6. Recommendations

As specified in ASTM D4609 [81], an effective soil stabilization scheme can be characterized as one that is able to produce a minimum improvement of 345 kPa in the mobilized UCS (in relation to the unstabilized scenario) [70,82,83,84]. With this in mind, none of the investigated SA-based mix designs were able to satisfy this requirement—that is, the maximum improvement for the three soil-GR blends containing 0%, 5% and 10% GR can be calculated as 230.8, 226.9 and 148.0 kPa (for the samples *R*_0_*S*_15_*T*_14_, *R*_5_*S*_15_*T*_14_ and *R*_10_*S*_15_*T*_14_), respectively. If the addition of GR is also considered as a physical soil-stabilizing factor, the maximum improvement can be calculated as 245.7 kPa (for the sample *R*_5_*S*_15_*T*_14_ in relation to *R*_0_*S*_0_*T*_0_), which is still less than the 345-kPa requirement. Assuming that the number of attraction sites—that is, the number of clay particles (or the soil clay content) and/or the amount of exchangeable divalent cations (i.e., Ca^2+^ and Mg^2+^)—available for the SA molecules was not exhausted with the employed SA dosages, higher SA dosages (greater than 15 g/L) may produce further improvements in the UCS. Nevertheless, beyond a certain SA (or polymer) dosage for which the available attraction sites are exhausted (or saturated), the clay particle flocculation process is expected to cease—beyond this critical dosage, the excess SA (or polymer) molecules will likely function as a lubricant rather than a flocculant, hence potentially reducing the mobilized UCS [39,41,54]. In view of the UCS results presented in this study, the maximum flocculation capacity (for the SA agent) may not have been achieved. As such, additional uniaxial compression tests employing higher SA dosages are recommended (particularly for GR-blended cases) to identify SA’s maximum stabilization capacity (hence re-examining the postulated clay–GR–SA amending mechanisms), and to recheck whether the 345-kPa requirement can be satisfied.

Furthermore, a systematically controlled test program should be carried out to examine the effects of other critical parameters representing real-life field conditions (or environmental fluctuations)—that is, mellowing time and compaction delay, curing temperature, relative humidity during curing, long-term curing (up to 90 days) performance and cyclic wetting–drying resistance—on the mechanical performance of compacted soil-GR–SA blends. Finally, it is worth mentioning that the leaching of heavy metals from the GR particles into the soil mass and hence water bodies (over time) could potentially raise some environmental concerns. Luckily, the majority of research studies in this context have shown that the degree of soil and water contamination both remain within the allowable limits suggested by various health and environmental agencies—a comprehensive review on this topic is given in Yadav and Tiwari [2]. More importantly, it can be argued that the potential risks involved with recycling and reusing discarded tires within our infrastructure system outweigh the long-term costs and hazards associated with traditional tire disposal techniques. In this context, additional permeability and leaching tests are recommended to further examine and better understand the potential risks involved with employing GR-based geomaterials in practice (as well as the potential role of SA in mitigating these risks).

## Figures and Tables

**Table 1:** Physical and mechanical properties of the test soil.

Soil Property	Value	Standard
**Particle-size distribution (PSD)**		
Sand fraction (2–4.75 mm) (%)	1	ASTM D422 [60]
Silt fraction (2–75 μm) (%)	47	ASTM D422 [60]
Clay fraction (<2 μm) (%)	52	ASTM D422 [60]
**Consistency/Atterberg limits**		
Liquid limit (LL) (%)	84.3	AS 1289.3.9.1 [61]
Plastic limit (PL) (%)	32.0	AS 1289.3.2.1 [62]
Plasticity index (PI) (%)	52.3	AS 1289.3.3.1 [63]
USCS soil classification	CH ^a^	ASTM D2487 [64]
**Compaction properties for standard Proctor energy (SPE)**		
Specific gravity of soil solids, $G_{s}^{S}$	2.73	ASTM D854 [65]
Optimum moisture content (OMC), *w*_opt_ (%) ^b^	28.0	ASTM D698 [66]
Maximum dry unit weight (MDUW), *γ*_dmax_ (kN/m^3^) ^b^	14.6	ASTM D698 [66]
Void ratio at MDUW, *e* ^c^	0.834	ASTM D698 [66]

^a^ Clay with high plasticity; ^b^ Tested under the standard Proctor energy (SPE) level and ^c^ Calculated as *e* = $G_{s}^{S}$*γ*_w_/*γ*_dmax_ − 1 (where *γ*_w_ = unit weight of water).

**Table 2:** Summary of the investigated soil-GR–SA mix designs and their properties.

Group	Sample	*f*_GR_ (%) ^a^	*D*_SA_ (g/L) ^b^	*T*_c_ (d) ^c^	*w*_o_ (%) ^d^	*γ*_do_ (kN/m^3^) ^e^	$G_{s}^{\mathrm{mix}}{\;}^{f}$	*e*_o_^g^
Untreated	*R*_0_ *S*_0_ *T*_0_	0	0	0	28.0	14.6	2.73	0.834
	*R*_5_ *S*_0_ *T*_0_	5			26.2	14.3	2.55	0.749
	*R*_10_ *S*_0_ *T*_0_	10			24.5	13.8	2.40	0.706
	*R*_20_ *S*_0_ *T*_0_	20			22.2	13.4	2.18	0.596
	*R*_30_ *S*_0_ *T*_0_	30			20.6	12.9	2.03	0.544
SA-treated	*R*_0_ *S*_5_ *T*_1,4,7,14_	0	5	1, 4, 7, 14	28.0	14.6	2.73	0.834
	*R*_0_ *S*_10_ *T*_1,4,7,14_		10					
	*R*_0_ *S*_15_ *T*_1,4,7,14_		15					
SA-treated	*R*_5_ *S*_5_ *T*_1,4,7,14_	5	5	1, 4, 7, 14	26.2	14.3	2.55	0.749
	*R*_5_ *S*_10_ *T*_1,4,7,14_		10					
	*R*_5_ *S*_15_ *T*_1,4,7,14_		15					
SA-treated	*R*_10_ *S*_5_ *T*_1,4,7,14_	10	5	1, 4, 7, 14	24.5	13.8	2.40	0.706
	*R*_10_ *S*_10_ *T*_1,4,7,14_		10					
	*R*_10_ *S*_15_ *T*_1,4,7,14_		15					

^a^ GR content, as per Equation (2); ^b^ SA dosage, as per Equation (3); ^c^ Curing duration; ^d^ Molding (as-compacted) MC; ^e^ Molding DUW; ^f^ Soil-GR mixture specific gravity, calculated by Equation (4); and ^g^ Molding void ratio, calculated as *e*_o_ = $G_{s}^{\mathrm{mix}}$*γ*_w_/*γ*_do_ − 1 (where *γ*_w_ = unit weight of water).

**Table 3:** Results of the SPE compaction and uniaxial compression tests for the untreated soil-GR blends—that is, *R*_0_*S*_0_*T*_0_, *R*_5_*S*_0_*T*_0_, *R*_10_*S*_0_*T*_0_, *R*_20_*S*_0_*T*_0_ and *R*_30_*S*_0_*T*_0_.

Sample	OMC, *w*_opt_ (%)	MDUW, *γ*_dmax_ (kN/m^3^)	UCS, *q*_u_ (kPa)	Improvement (%) ^a^
*R*_0_ *S*_0_ *T*_0_	28.0	14.6	127.6	—
*R*_5_ *S*_0_ *T*_0_	26.2	14.3	146.4	+15
*R*_10_ *S*_0_ *T*_0_	24.5	13.8	120.5	−6
*R*_20_ *S*_0_ *T*_0_	22.2	13.4	115.4	−10
*R*_30_ *S*_0_ *T*_0_	20.6	12.9	81.3	−36

^a^ Percent change in the UCS (or *q*_u_) in relation to the test soil (*R*_0_*S*_0_*T*_0_).

## Data Availability

The data that support the findings of this study are available from the corresponding author upon reasonable request.

## Abbreviations

AS	Australian Standard
ASTM	American Society for Testing and Materials
CH	Clay with high plasticity
CV	Coefficient of variation
DUW	Dry unit weight
ELT	End-of-life tire
GR	Ground rubber
LL	Liquid limit
MC	Moisture content
MDUW	Maximum dry unit weight
OMC	Optimum moisture content
PI	Plasticity index
PL	Plastic limit
PSD	Particle-size distribution
*R_x_*	*x*% Ground rubber
SA	Sodium alginate
SD	Standard deviation
SEM	Scanning electron microscopy
SGF	Strength-gain-factor
SP	Poorly-graded sand
SPE	Standard Proctor energy
*S_y_*	*y* g/L Sodium alginate
*T_z_*	*z* days of curing
UCS	Uniaxial compressive strength
USCS	Unified Soil Classification System

## Notations

*C*_c_	Coefficient of curvature
*C*_u_	Coefficient of curvature
*d*	Particle diameter (in mm)
*d*_10_	Particle diameter corresponding to 10% finer (in mm)
*d*_30_	Particle diameter corresponding to 30% finer (in mm)
*d*_50_	Particle diameter corresponding to 50% finer (in mm)
*d*_60_	Particle diameter corresponding to 60% finer (in mm)
*d*_90_	Particle diameter corresponding to 90% finer (in mm)
*D*_SA_	Sodium alginate dosage (in g/L)
*e*	Void ratio
*e*_o_	Moulding (as-compacted) void ratio
*f*_GR_	Ground rubber content (in %)
$G_{s}^{\mathrm{GR}}$	Specific gravity of ground rubber particles
$G_{s}^{\mathrm{mix}}$	soil-GRound rubber mixture specific gravity
$G_{s}^{S}$	Specific gravity of soil solids
*M*_DS_	Mass of oven-dried soil (in g)
*M*_GR_	Mass of ground rubber (in g)
*M*_SA_	Mass of sodium alginate powder (in g)
*q*_u_	Uniaxial compressive strength (in kPa)
$q_{u}^{T}$	Uniaxial compressive strength of SA-treated soil-GR samples (in kPa)
$q_{u}^{U}$	Uniaxial compressive strength of untreated soil-GR samples (in kPa)
*T*_c_	Curing duration (in d)
*V*_DW_	Volume of distilled water (in L)
*w*	Moisture content (in %)
*w*_o_	Molding (as-compacted) moisture content (in %)
*w*_opt_	Optimum moisture content (in %)
*γ*_d_	Dry unit weight (in kN/m^3^)
*γ*_dmax_	Maximum dry unit weight (in kN/m^3^)
*γ*_do_	Molding (as-compacted) dry unit weight (in kN/m^3^)
*γ*_w_	Unit weight of water (in kN/m^3^)

## References

[1] Czajczyńska D., Krzyżyńska R., Jouhara H., Spencer N. (2017). Use of pyrolytic gas from waste tire as a fuel: A review. Energy.

[2] Yadav J.S., Tiwari S.K. (2019). The impact of end-of-life tires on the mechanical properties of fine-grained soil: A review. Environ. Dev. Sustain..

[3] Tian X., Zhuang Q., Han S., Li S., Liu H., Li L., Zhang J., Wang C., Bian H. (2021). A novel approach of reapplication of carbon black recovered from waste tyre pyrolysis to rubber composites. J. Clean. Prod..

[4] Soltani A., Deng A., Taheri A., Mirzababaei M., Nikraz H. (2019). Interfacial shear strength of rubber-reinforced clays: A dimensional analysis perspective. Geosynth. Int..

[5] Raeesi R., Soltani A., King R., Disfani M.M. (2020). Field performance monitoring of waste tire-based permeable pavements. Transp. Geotech..

[6] Soltani A., Taheri A., Deng A., O’Kelly B.C. (2020). Improved geotechnical behavior of an expansive soil amended with tire-derived aggregates having different gradations. Minerals.

[7] Patil U., Valdes J.R., Evans T.M. (2011). Swell mitigation with granulated tire rubber. J. Mater. Civ. Eng..

[8] Trouzine H., Bekhiti M., Asroun A. (2012). Effects of scrap tyre rubber fibre on swelling behaviour of two clayey soils in Algeria. Geosynth. Int..

[9] Srivastava A., Pandey S., Rana J. (2014). Use of shredded tyre waste in improving the geotechnical properties of expansive black cotton soil. Geomech. Geoengin..

[10] Signes C.H., Garzón-Roca J., Fernández P.M., de la Torre M.E.G., Franco R.I. (2016). Swelling potential reduction of Spanish argillaceous marlstone Facies Tap soil through the addition of crumb rubber particles from scrap tyres. Appl. Clay Sci..

[11] Yadav J.S., Tiwari S.K. (2018). Influence of crumb rubber on the geotechnical properties of clayey soil. Environ. Dev. Sustain..

[12] Mukherjee K., Mishra A.K. (2019). Hydraulic and mechanical characteristics of compacted sand–bentonite: Tyre chips mix for its landfill application. Environ. Dev. Sustain..

[13] Soltani A., Deng A., Taheri A., Sridharan A. (2019). Swell–shrink–consolidation behavior of rubber-reinforced expansive soils. Geotech. Test. J..

[14] Soltani A., Deng A., Taheri A., Mirzababaei M., Vanapalli S.K. (2019). Swell–shrink behavior of rubberized expansive clays during alternate wetting and drying. Minerals.

[15] Abbaspour M., Narani S.S., Aflaki E., Moghadas Nejad F., Mir Mohammad Hosseini S.M. (2020). Strength and swelling properties of a waste tire textile fiber-reinforced expansive soil. Geosynth. Int..

[16] Li S., Li D. (2018). Mechanical properties of scrap tire crumbs–clayey soil mixtures determined by laboratory tests. Adv. Mater. Sci. Eng..

[17] Indraratna B., Qi Y., Ngo T.N., Rujikiatkamjorn C., Neville T., Ferreira F.B., Shahkolahi A. (2019). Use of geogrids and recycled rubber in railroad infrastructure for enhanced performance. Geosciences.

[18] Saberian M., Li J. (2019). Long-term permanent deformation behaviour of recycled concrete aggregate with addition of crumb rubber in base and sub-base applications. Soil Dyn. Earthq. Eng..

[19] Tsiavos A., Alexander N.A., Diambra A., Ibraim E., Vardanega P.J., Gonzalez-Buelga A., Sextos A. (2019). A sand–rubber deformable granular layer as a low-cost seismic isolation strategy in developing countries: Experimental investigation. Soil Dyn. Earthq. Eng..

[20] Akbarimehr D., Fakharian K. (2021). Dynamic shear modulus and damping ratio of clay mixed with waste rubber using cyclic triaxial apparatus. Soil Dyn. Earthq. Eng..

[21] Akbulut S., Arasan S., Kalkan E. (2007). Modification of clayey soils using scrap tire rubber and synthetic fibers. Appl. Clay Sci..

[22] Cabalar A.F., Karabash Z., Mustafa W.S. (2014). Stabilising a clay using tyre buffings and lime. Road Mater. Pavement Des..

[23] Tajdini M., Nabizadeh A., Taherkhani H., Zartaj H. (2017). Effect of added waste rubber on the properties and failure mode of kaolinite clay. Int. J. Civ. Eng..

[24] Irani N., Ghasemi M. (2019). Effect of scrap tyre on strength properties of untreated and lime-treated clayey sand. Eur. J. Environ. Civ. Eng..

[25] Soltani A., Taheri A., Deng A., Nikraz H. (2020). Tyre rubber and expansive soils: Two hazards, one solution. Proc. Inst. Civ. Eng. Constr. Mater..

[26] Yang Z., Zhang Q., Shi W., Lv J., Lu Z., Ling X. (2020). Advances in properties of rubber reinforced soil. Adv. Civ. Eng..

[27] Kalkan E. (2013). Preparation of scrap tire rubber fiber–silica fume mixtures for modification of clayey soils. Appl. Clay Sci..

[28] Bekhiti M., Trouzine H., Rabehi M. (2019). Influence of waste tire rubber fibers on swelling behavior, unconfined compressive strength and ductility of cement stabilized bentonite clay soil. Constr. Build. Mater..

[29] Yadav J.S., Garg A., Tiwari S.K. (2021). Strength and ductility behaviour of rubberised cemented clayey soil. Proc. Inst. Civ. Eng. Gr. Improv..

[30] Williamson S., Cortes D.D. (2014). Dimensional analysis of soil–cement mixture performance. Géotechnique Lett..

[31] Georgees R.N., Hassan R.A., Evans R.P., Jegatheesan P. (2015). Effect of the use of a polymeric stabilizing additive on unconfined compressive strength of soils. Transp. Res. Rec. J. Transp. Res. Board.

[32] Chang I., Lee M., Tran A.T.P., Lee S., Kwon Y.M., Im J., Cho G.C. (2020). Review on biopolymer-based soil treatment (BPST) technology in geotechnical engineering practices. Transp. Geotech..

[33] Estabragh A.R., Beytolahpour I., Javadi A.A. (2011). Effect of resin on the strength of soil–cement mixture. J. Mater. Civ. Eng..

[34] Khatami H.R., O’Kelly B.C. (2013). Improving mechanical properties of sand using biopolymers. J. Geotech. Geoenviron. Eng..

[35] Chang I., Im J., Cho G.C. (2016). Introduction of microbial biopolymers in soil treatment for future environmentally-friendly and sustainable geotechnical engineering. Sustainability.

[36] Muguda S., Booth S.J., Hughes P.N., Augarde C.E., Perlot C., Bruno A.W., Gallipoli D. (2017). Mechanical properties of biopolymer-stabilised soil-based construction materials. Géotechnique Lett..

[37] He S., Yu X., Banerjee A., Puppala A.J. (2018). Expansive soil treatment with liquid ionic soil stabilizer. Transp. Res. Rec. J. Transp. Res. Board.

[38] Mirzababaei M., Arulrajah A., Horpibulsuk S., Soltani A., Khayat N. (2018). Stabilization of soft clay using short fibers and poly vinyl alcohol. Geotext. Geomembr..

[39] Soltani A., Deng A., Taheri A., O’Kelly B.C. (2019). Engineering reactive clay systems by ground rubber replacement and polyacrylamide treatment. Polymers.

[40] Ghasemzadeh H., Mehrpajouh A., Pishvaei M., Mirzababaei M. (2020). Effects of curing method and glass transition temperature on the unconfined compressive strength of acrylic liquid polymer-stabilized kaolinite. J. Mater. Civ. Eng..

[41] Soltani A., Raeesi R., O’Kelly B.C. (2020). Cyclic swell–shrink behavior of an expansive soil treated with a sulfonated oil. Proc. Inst. Civ. Eng. Gr. Improv..

[42] Theng B. (1982). Clay–polymer interactions: Summary and perspectives. Clay Clay Miner..

[43] Ben-Hur M., Malik M., Letey J., Mingelgrin U. (1992). Adsorption of polymers on clays as affected by clay charge and structure, polymers properties, and water quality. Soil Sci..

[44] Letey J. (1994). Adsorption and desorption of polymers on soil. Soil Sci..

[45] Slaný M., Jankovič Ľ., Madejová J. (2019). Structural characterization of organo-montmorillonites prepared from a series of primary alkylamines salts: Mid-IR and near-IR study. Appl. Clay Sci..

[46] Johnson F.A., Craig D.Q.M., Mercer A.D. (1997). Characterization of the block structure and molecular weight of sodium alginates. J. Pharm. Pharmacol..

[47] George M., Abraham T.E. (2006). Polyionic hydrocolloids for the intestinal delivery of protein drugs: Alginate and chitosan—A review. J. Control. Release.

[48] Kulkarni R.V., Sreedhar V., Mutalik S., Setty C.M., Sa B. (2010). Interpenetrating network hydrogel membranes of sodium alginate and poly(vinyl alcohol) for controlled release of prazosin hydrochloride through skin. Int. J. Biol. Macromol..

[49] Pignolet L.H., Waldman A.S., Schechinger L., Govindarajoo G., Nowick J.S., Labuza T. (1998). The alginate demonstration: Polymers, food science, and ion exchange. J. Chem. Educ..

[50] El-Aassar M.R., Hafez E.E., El-Deeb N.M., Fouda M.M.G. (2014). Microencapsulation of lectin anti-cancer agent and controlled release by alginate beads, biosafety approach. Int. J. Biol. Macromol..

[51] Qin Y., Qin Y. (2018). Seaweed hydrocolloids as thickening, gelling, and emulsifying agents in functional food products. Bioactive Seaweeds for Food Applications: Natural Ingredients for Healthy Diets.

[52] Bouazza A., Gates W.P., Ranjith P.G. (2009). Hydraulic conductivity of biopolymer-treated silty sand. Géotechnique.

[53] Peng C., Zheng J., Huang S., Li S., Li D., Cheng M., Liu Y. (2017). Application of sodium alginate in induced biological soil crusts: Enhancing the sand stabilization in the early stage. J. Appl. Phycol..

[54] Arab M.G., Mousa R.A., Gabr A.R., Azam A.M., El-Badawy S.M., Hassan A.F. (2019). Resilient behavior of sodium alginate-treated cohesive soils for pavement applications. J. Mater. Civ. Eng..

[55] Fatehi H., Bahmani M., Noorzad A., Meehan C.L., Kumar S., Pando M.A., Coe J.T. (2019). Strengthening of dune sand with sodium alginate biopolymer. Geo-Congress 2019: Soil Improvement (GSP 309).

[56] Almajed A., Lemboye K., Arab M.G., Alnuaim A. (2020). Mitigating wind erosion of sand using biopolymer-assisted EICP technique. Soils Found..

[57] Soldo A., Miletić M., Auad M.L. (2020). Biopolymers as a sustainable solution for the enhancement of soil mechanical properties. Sci. Rep..

[58] Zhao Y., Zhuang J., Wang Y., Jia Y., Niu P., Jia K. (2020). Improvement of loess characteristics using sodium alginate. Bull. Eng. Geol. Environ..

[59] Lemboye K., Almajed A., Alnuaim A., Arab M., Alshibli K. (2021). Improving sand wind erosion resistance using renewable agriculturally derived biopolymers. Aeolian Res..

[60] ASTM D422 (2007). Standard Test. Method for Particle-Size Analysis of Soils.

[61] AS 1289.3.9.1 (2015). Methods of Testing Soils for Engineering Purposes: Soil Classification Tests—Determination of the Cone Liquid Limit of a Soil.

[62] AS 1289.3.2.1 (2009). Methods of Testing Soils for Engineering Purposes: Soil Classification Tests—Determination of the Plastic Limit of a Soil—Standard Method.

[63] AS 1289.3.3.1 (2009). Methods of Testing Soils for Engineering Purposes: Soil Classification Tests—Calculation of the Plasticity Index of a Soil.

[64] ASTM D2487 (2017). Standard Practice for Classification of Soils for Engineering Purposes (Unified Soil Classification System).

[65] ASTM D854 (2014). Standard Test. Methods for Specific Gravity of Soil Solids by Water Pycnometer.

[66] ASTM D698 (2012). Standard Test. Methods for Laboratory Compaction Characteristics of Soil Using Standard Effort (12 400 ft-lbf/ft^3^ (600 kN-m/m^3^)).

[67] Yadav J.S., Tiwari S.K. (2017). Effect of waste rubber fibres on the geotechnical properties of clay stabilized with cement. Appl. Clay Sci..

[68] Soltani A., Deng A., Taheri A., Sridharan A. (2019). Consistency limits and compaction characteristics of clay soils containing rubber waste. Proc. Inst. Civ. Eng. Geotech. Eng..

[69] Estabragh A.R., Javadi A.A. (2008). Critical state for overconsolidated unsaturated silty soil. Can. Geotech. J..

[70] Zhang J., Soltani A., Deng A., Jaksa M.B. (2019). Mechanical performance of jute fiber-reinforced micaceous clay composites treated with ground-granulated blast-furnace slag. Materials.

[71] Zhang J., Soltani A., Deng A., Jaksa M.B. (2019). Mechanical behavior of micaceous clays. J. Rock Mech. Geotech. Eng..

[72] ASTM D2166 (2016). Standard Test. Method for Unconfined Compressive Strength of Cohesive Soil.

[73] Madejová J., Barlog M., Jankovič Ľ., Slaný M., Pálková H. (2021). Comparative study of alkylammonium- and alkylphosphonium-based analogues of organo-montmorillonites. Appl. Clay Sci..

[74] Yaghoubi M., Shukla S.K., Mohyeddin A. (2018). Effects of addition of waste tyre fibres and cement on the engineering behaviour of Perth sand. Geomech. Geoengin..

[75] Elsayed E.M., Elnouby M.S., Gouda M.H., Elessawy N.A., Santos D.M.F. (2020). Effect of the morphology of tungsten oxide embedded in sodium alginate/polyvinylpyrrolidone composite beads on the photocatalytic degradation of methylene blue dye solution. Materials.

[76] Latifi N., Rashid A.S.A., Siddiqua S., Majid M.Z.A. (2016). Strength measurement and textural characteristics of tropical residual soil stabilised with liquid polymer. Measurement.

[77] Ouwerx C., Velings N., Mestdagh M.M., Axelos M.A.V. (1998). Physico-chemical properties and rheology of alginate gel beads formed with various divalent cations. Polym. Gels Netw..

[78] Zhang Y., Yu W., Lv G., Zhu J., Wang W., Ma X., Liu X., Young M.M. (2011). The artificial organ: Cell encapsulation. Reference Module in Biomedical Sciences: Comprehensive Biotechnology (Second Edition).

[79] Valapa R.B., Loganathan S., Pugazhenthi G., Thomas S., Varghese T.O., Jlassi K., Chehimi M.M., Thomas S. (2017). An overview of polymer–clay nanocomposites. Clay–Polymer Nanocomposites.

[80] Soltani A., Deng A., Taheri A., Mirzababaei M. (2018). Rubber powder–polymer combined stabilization of South Australian expansive soils. Geosynth. Int..

[81] ASTM D4609 (2008). Standard Guide for Evaluating Effectiveness of Admixtures for Soil Stabilization.

[82] Iyengar S.R., Masad E., Rodriguez A.K., Bazzi H.S., Little D., Hanley H.J.M. (2013). Pavement subgrade stabilization using polymers: Characterization and performance. J. Mater. Civ. Eng..

[83] Shahbazi M., Rowshanzamir M., Abtahi S.M., Hejazi S.M. (2017). Optimization of carpet waste fibers and steel slag particles to reinforce expansive soil using response surface methodology. Appl. Clay Sci..

[84] Rodriguez A.K., Ayyavu C., Iyengar S.R., Bazzi H.S., Masad E., Little D., Hanley H.J.M. (2018). Polyampholyte polymer as a stabiliser for subgrade soil. Int. J. Pavement Eng..

